# Correction: Nitric oxide/paclitaxel micelles enhance anti-liver cancer effects and paclitaxel sensitivity by inducing ferroptosis, endoplasmic reticulum stress and pyroptosis

**DOI:** 10.1039/d3ra90117c

**Published:** 2023-11-30

**Authors:** Huilan Li, Xiaoyu Deng, Ziwei Zhang, Zunhua Yang, Hesong Huang, Xide Ye, Lingyun Zhong, Guoliang Xu, Ronghua Liu, Yuanying Fang

**Affiliations:** a National Engineering Research Center for Manufacturing Technology of TCM Solid Preparation, Jiangxi University of Chinese Medicine Nanchang 330006 China fangyuanying@163.com; b College of Pharmacy, Jiangxi University of Chinese Medicine Nanchang 330004 China 20070994@jxutcm.edu.cn

## Abstract

Correction for ‘Nitric oxide/paclitaxel micelles enhance anti-liver cancer effects and paclitaxel sensitivity by inducing ferroptosis, endoplasmic reticulum stress and pyroptosis’ by Huilan Li *et al.*, *RSC Adv.*, 2023, **13**, 31772–31784, https://doi.org/10.1039/D3RA04861F.

The authors regret that the name of one of the authors (Lingyun Zhong) was shown incorrectly in the original article. The corrected author list is as shown above.

The Royal Society of Chemistry apologises for these errors and any consequent inconvenience to authors and readers.

## Supplementary Material

